# The Respiratory Commensal Bacterium *Dolosigranulum pigrum* 040417 Improves the Innate Immune Response to *Streptococcus pneumoniae*

**DOI:** 10.3390/microorganisms9061324

**Published:** 2021-06-18

**Authors:** Fernanda Raya Tonetti, Mikado Tomokiyo, Ramiro Ortiz Moyano, Sandra Quilodrán-Vega, Hikari Yamamuro, Paulraj Kanmani, Vyacheslav Melnikov, Shoichiro Kurata, Haruki Kitazawa, Julio Villena

**Affiliations:** 1 Laboratory of Immunobiotechnology, Reference Centre for Lactobacilli (CERELA-CONICET), Tucumán 4000, Argentina; frayatonetti@gmail.com (F.R.T.); rortiz@cerela.org.ar (R.O.M.); 2 Food and Feed Immunology Group, Laboratory of Animal Food Function, Graduate School of Agricultural Science, Tohoku University, Sendai 980-8572, Japan; mikado0403@gmail.com (M.T.); hikari.yamamuro.r5@dc.tohoku.ac.jp (H.Y.); kanmanibiotech2007@gmail.com (P.K.); 3 Livestock Immunology Unit, International Education and Research Center for Food and Agricultural Immunology (CFAI), Graduate School of Agricultural Science, Tohoku University, Sendai 980-8572, Japan; 4 Laboratory of Food Microbiology, Faculty of Veterinary Sciences, University of Concepción, Chillán 3780000, Chile; squilodran@udec.cl; 5 Gabrichevsky Research Institute for Epidemiology and Microbiology, 125212 Moscow, Russia; goutch@mail.ru; 6 Laboratory of Molecular Genetics, Graduate School of Pharmaceutical Sciences, Tohoku University, Sendai 980-8578, Japan; kurata@mail.pharm.tohoku.ac.jp

**Keywords:** respiratory commensal bacteria, upper respiratory tract, next-generation probiotics, *Dolosigranulum pigrum*, *Streptococcus pneumoniae*, alveolar macrophages

## Abstract

Previously, we demonstrated that the nasal administration of *Dolosigranulum pigrum* 040417 differentially modulated the respiratory innate immune response triggered by the activation of Toll-like receptor 2 in infant mice. In this work, we aimed to evaluate the beneficial effects of *D. pigrum* 040417 in the context of *Streptococcus pneumoniae* infection and characterize the role of alveolar macrophages (AMs) in the immunomodulatory properties of this respiratory commensal bacterium. The nasal administration of *D. pigrum* 040417 to infant mice significantly increased their resistance to pneumococcal infection, differentially modulated respiratory cytokines production, and reduced lung injuries. These effects were associated to the ability of the 040417 strain to modulate AMs function. Depletion of AMs significantly reduced the capacity of the 040417 strain to improve both the reduction of pathogen loads and the protection against lung tissue damage. We also demonstrated that the immunomodulatory properties of *D. pigrum* are strain-specific, as *D. pigrum* 030918 was not able to modulate respiratory immunity or to increase the resistance of mice to an *S. pneumoniae* infection. These findings enhanced our knowledge regarding the immunological mechanisms involved in modulation of respiratory immunity induced by beneficial respiratory commensal bacteria and suggested that particular strains could be used as next-generation probiotics.

## 1. Introduction

Different microbial species colonize the upper respiratory tract (URT) after birth. The variety of species that are found in the respiratory mucosa depends on several factors including the mode of delivery, the environment, and the interaction of these microbial communities with the immune system [1]. The establishment of a stable respiratory microbiota has been associated to some effects beneficial for the host. It was documented that the URT microbiota prevents pathogen colonization by obstructing adhesion sites, enhancing pathogen clearance by competition for nutrients, or production of antimicrobial substances as well as modulating the host immune responses [2,3]. Alterations in the composition or function of the respiratory microbiota—due, for example, to the use of antibiotics—can induce reduction or loss of the beneficial members that help in the protection of the respiratory tract against opportunistic pathogens [4]. Pathogens such as *Streptococcus pneumoniae* may then take advantage of the disturbance in the respiratory microbiota to grow and spread, leading to local or systemic acute infections, such as acute otitis media, pneumonia, septicaemia, or meningitis [5].

Although the nasopharyngeal microbiota has been less studied than the intestinal microbiota, recent reports have suggested that certain members of this microbial population may positively influence the host respiratory health [6]. In this regard, recent research has identified the species *Corynebacterium* and *Dolosigranulum* as important beneficial members of the nasopharynx microbiota [1,3,7,8,9]. The advances in the knowledge of the benefits conferred to the host by members of these respiratory commensal bacteria species and the better understanding of their mechanisms of action made it possible to propose them as next-generation probiotics for the respiratory tract [2,10,11,12,13,14].

Among the URT commensal bacterial species that have gained interest as potential next-generation probiotics, *Dolosigranulum pigrum* is one of the most promising to beneficially influence human respiratory health. It was observed that this Gram-positive, catalase-negative *Firmicute* bacterium is abundant in healthy URT, reaching a relative abundance of up to 50% [15], and different studies have related it to vaginal delivery and breastfeeding [16]. A lower incidence of URT infections and a reduced probability of contracting bronchiolitis were observed in children who had higher numbers of *D. pigrum* in their respiratory microbiota [17]. Moreover, *D. pigrum* seems to reduce the risk of acquiring respiratory infections, such as the invasive disease caused by *S. pneumoniae* [7] or flu by the influenza virus [18].

We previously demonstrated that the nasal administration of *D. pigrum* differentially modulates the respiratory immune response triggered by the activation of Toll-like receptor (TLR)-2 or TLR-3 in infant mice, increasing the resistance to primary respiratory syncytial virus (RSV) and pneumococcal infections [12]. Interestingly, a strain-dependent immunomodulatory effect was observed in our studies. While *D. pigrum* 040417 was capable of decreasing RSV titres and pneumococcal cell counts in lungs of infected mice, *D. pigrum* 030918 was not able to induce modifications when compared with control infected animals. In this work, we aimed to further advance the characterization of the beneficial effects of *D. pigrum* 040417 by analysing its impact on the respiratory immune response triggered by *S. pneumoniae* infection. Our studies focused on the role of alveolar macrophages (AMs) in the immunomodulatory properties of the 040417 strain in the context of pneumococcal infection. Moreover, studies using the non-immunomodulatory *D. pigrum* 030918 strain were performed for comparison.

## 2. Materials and Methods

### 2.1. Microorganisms

*Dolosigranulum pigrum* 040417 and *D. pigrum* 030918 were cultured at 37 °C for 18 h (late log phase) in trypticase soy broth. Bacterial suspensions were prepared as previously described [12]. Briefly, bacteria were harvested by centrifugation at 3000× *g* for 10 min, washed three times with sterile 0.01 M phosphate buffer saline (PBS, pH 7.2), and resuspended in sterile PBS.

*Streptococcus pneumoniae* serotypes 6B, 14, and 3 (ANLIS, Buenos Aires, Argentina) were cultured on blood agar for 18 h. Colonies were suspended in Todd Hewitt broth (Oxoid, Cambridge, UK) supplemented with 0.5% yeast extract and incubated overnight at 37 °C. Bacteria cells were harvested and washed three times with sterile PBS. Cell density was adjusted to 1 × 10^9^ CFU/mL. Serial dilutions and quantitative subcultures were performed on blood agar to confirm the size of the inoculum used in the challenge experiments as described previously [19].

### 2.2. Animals and Experimental Infection

Three weeks old BALB/c mice were obtained from the closed colony kept at CERELA (San Miguel de Tucumán, Argentina). During the experiments, they were individually housed in plastic cages at room temperature. *D. pigrum* 040417 or *D. pigrum* 030918 were nasally administered to different groups of mice for 5 consecutive days at a dose of 10^8^ cells/mouse/day in 50 µL of PBS. Control animals only received 50 µL of PBS. The treated groups and the untreated control mice were fed a conventional balanced diet ad libitum. All experiments were approved by the Ethical Committee for Animal Care of Reference Centre for Lactobacilli (CERELA-CONICET, Tucuman, Argentina) and all efforts were made to minimize suffering. No signs of discomfort or pain and no deaths were observed before mice reached the endpoints. One day after the last administration of viable bacteria, mice were nasally challenged with 10^6^ CFU of the pathogen (*S. pneumoniae* serotype 6B, 14, or 3) suspended in 20 µL of PBS. The inoculum was inhaled involuntarily and to facilitate its migration to the alveoli, mice were held in an upright position with their heads up for 2 min. Mice were sacrificed at different time points after the challenge. The lungs were removed to determine *S. pneumoniae* counts. Lungs were weighted, homogenized, and appropriately diluted in sterile peptone water. The dilutions were plated in duplicate on blood agar and incubated for 18 h at 37 °C. *S. pneumoniae* was identified using standard techniques and the results were expressed as logarithm of CFU/g of lung. Blood samples were used to perform haemocultures that were expressed as positive or negative.

### 2.3. Broncho-Alveolar Lavage (BAL) Sampling

The BAL samples were obtained as previously described [20]. Briefly, the trachea was exposed and intubated with a catheter and two washes of the lungs were performed with sterile PBS. Then, the wash obtained was centrifuged and the cell-free supernatants were kept at −70 °C until analysis.

### 2.4. Lung Tissue Injury Studies

The albumin content in BAL was quantified as an indicator of the increased permeability of the bronchoalveolar capillary barrier [21]. In addition, lactate dehydrogenase (LDH) activity was quantified as an indicator of general cytotoxicity [22]. Albumin content was determined colorimetrically using a diagnostic kit (Wiener Lab, Buenos Aires, Argentina) that is based on the binding of albumin to bromocresol green. LDH activity, expressed as units per litre of BAL fluid, was determined using Wiener’s reagents and procedures (Wiener Lab), which measure the formation of the reduced form of nicotinamide adenine dinucleotide (NAD).

### 2.5. Determination of Cell Populations

The total number of BAL leukocytes was determined using a haemocytometer. Differential cell counts in BAL were obtained by microscopically counting cells in smears stained with May-Grunwald-Giemsa as described before [20].

### 2.6. Primary Cultures of Alveolar Macrophages

Primary cultures of murine AMs were performed as previously described [21]. Briefly, BAL macrophages from control mice and from animals treated with *D. pigrum* 040417 or *D. pigrum* 030918 were obtained using 1 mL of warm sterile PBS containing 5 mM EDTA. The AMs were transferred to new sterile tubes, washed twice in sterile PBS and resuspended in RPMI 1640 medium with 10% FBS, 1 mM l-glutamine, and 100 U/mL penicillin–streptomycin. The cells were seeded in 24-well plates at a density of 10^5^ cells/well and incubated for 2 h at 37 °C in 5% CO_2_ to promote adherence. Washes were carried out to eliminate non-adherent cells and the AMs were maintained in culture in RPMI 1640 medium with 10% FBS, 1 mM l-glutamine, and 100 U/mL of penicillin–streptomycin at 37 °C in 5% CO_2_ for 24 h before stimulation. AMs were stimulated in vitro with *S. pneumoniae* serotype 3. Supernatants were collected before (basal conditions) and 24 h after challenge for cytokine analysis.

### 2.7. Cytokine Concentrations in BAL and Culture Supernatants

The concentrations of tumour necrosis factor (TNF)-α, interferon (IFN)-γ, IFN-β, interleukin (IL)-6, IL-1β, IL-27, IL-17, and IL-10 were measured with a commercially available enzyme-linked immunosorbent assay (ELISA) technique kits following the manufacturer’s recommendations (R&D Systems, Minneapolis, MN, USA). The chemokine (C–C motif) ligand 2 (CCL2) concentration was measured with commercially available ELISA technique kits following the manufacturer’s recommendations (Abcam, Cambridge, UK).

### 2.8. Analysis of Alveolar Macrophages by Flow Cytometry

A single cell suspension was obtained from lung samples as previously described [11]. Briefly, the lungs were removed and then finely minced. Samples were then incubated for 90 min with 300 U collagenase (Yakult Honsha Co., Tokyo, Japan) in 15 mL of RPMI 1640 medium (Sigma, Tokyo, Japan). The lungs treated with this enzyme were tapped on a plastic plate to dissociate the tissue into individual cells. Erythrocytes were depleted by hypotonic lysis and cells were washed with RPMI medium supplemented with 10% heat inactivated foetal calf serum. Finally, cells were counted using trypan blue exclusion and then resuspended at an appropriate concentration of 5 × 10^6^ cells/mL.

Pulmonary cell suspensions were preincubated with mouse anti-CD32/CD16 monoclonal antibodies (Fc block) for 15 min at 4 °C and washed with FACS buffer. The cells were then stained with fluorochrome-conjugated antibodies against CD11c (APC), SiglecF (PE) (BD Bioscience, San José, CA, USA), CD45 (FITC) (eBioscience, San Diego, CA, USA) and MHC-II (PerCP) (Thermo Fisher Scientific, Waltham, MA, USA). The cells were then analysed on a BD FACSCalibur™ flow cytometer (BD Biosciences, Franklin Lakes, NJ, USA) and the data were analysed with FlowJo software (TreeStar, Franklin Lakes, NJ, USA). The total number of cells in each population was determined by multiplying the percentages of subsets within a series of negative or positive markers by the total number of cells determined for each tissue sample [11].

### 2.9. In Vivo Depletion of Alveolar Macrophages

To deplete AMs, liposomes containing clodronate (dichloromethylene bisphosphonate) (CLP; Clophosome, Stratech, UK) were used. Mice were inoculated intranasally with 50 µL of CLP for two consecutive days, as previously described [21]. Mice were then stimulated with *D. pigrum* 040417 and challenged with *S. pneumoniae* according to the scheme described above. An equal treatment with empty liposomes (ELP) served as control.

### 2.10. Statistical Analysis

Experiments were made in triplicate and results were expressed as mean ± standard deviation (SD). Normal distributed data were tested by two-way ANOVA. Tukey’s test (for pairwise comparisons of the means) or the Fisher’s least significant difference (LSD) test (for multi-comparison) was used to evaluate the differences among groups. Differences were considered significant at *p* < 0.05.

## 3. Results

### 3.1. The Respiratory Commensal Bacteria D. pigrum Improve Resistance to Pneumococcal Infection in a Strain-Dependent Manner

**Figure 1:**
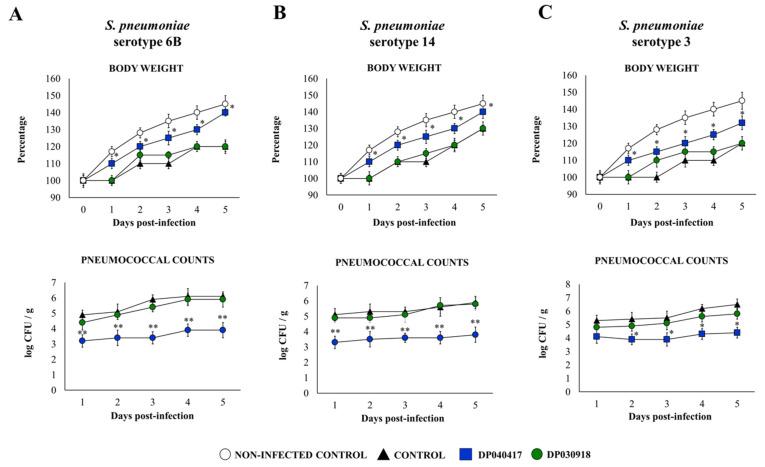
Effect of the respiratory commensal bacteria *Dolosigranulum pigrum* on resistance to *Streptococcus pneumoniae* infection. Infant mice were nasally primed with viable *D. pigrum* (DP) strain 040417 or 030918 during five consecutive days at a dose of 10^8^ bacterial cells/mouse/day. One day after the last administration of DP strains, mice were challenged with (**A**) *S. pneumoniae* serotypes 6B (**A**), 14 (**B**), or 3 (**C**). Non-treated infant mice challenged with the corresponding serotypes of *S. pneumoniae* were used as controls. Changes in body weight and lung pneumococcal cell counts were evaluated on different time points after the bacterial challenge. The results represent data from three independent experiments (*n* = 6 per each group at each time point). Significantly different when compared with control at the same time point, * *p* < 0.05 or ** *p* < 0.01.

We first aimed to evaluate if different strains of the respiratory commensal bacteria belonging to the species *D. pigrum* were able to influence the resistance of infant mice to the primary infection with *S. pneumoniae*. For this purpose, infant mice were nasally primed with viable *D. pigrum* strains 030918 or 040417 for five consecutive days. One day after the last administration of the respiratory commensal bacteria, mice were challenged with different *S. pneumoniae* serotypes including 6B, 14, or 3 (Figure 1).

The challenge of infant mice with *S. pneumoniae* induced a delay in the body weight gain of infected control mice in comparison with the mice treated with *D. pigrum* 040417 (Figure 1). In fact, the 040417 strain significantly improved the body weight gain of infant mice after the challenge with the three serotypes of the bacterial pathogen, although the mice in this group did not reach the percentages of non-infected controls. In contrast, body weight changes in the mice treated with *D. pigrum* 030918 were not different from infected controls (Figure 1). All the pneumococcal serotypes were detected in the lungs (Figure 1) and blood (data not shown) of all experimental groups during the 5 days studied. However, the *D. pigrum* 040417 treatment was able to significantly reduce bacterial replication in the respiratory tract compared with controls (Figure 1). In addition, the 040417 strain prevented the dissemination of pneumococci into the blood (data not shown). Lung pneumococcal counts (Figure 1) and haemocultures in the mice treated with *D. pigrum* 030918 were not different from controls.

**Figure 2:**
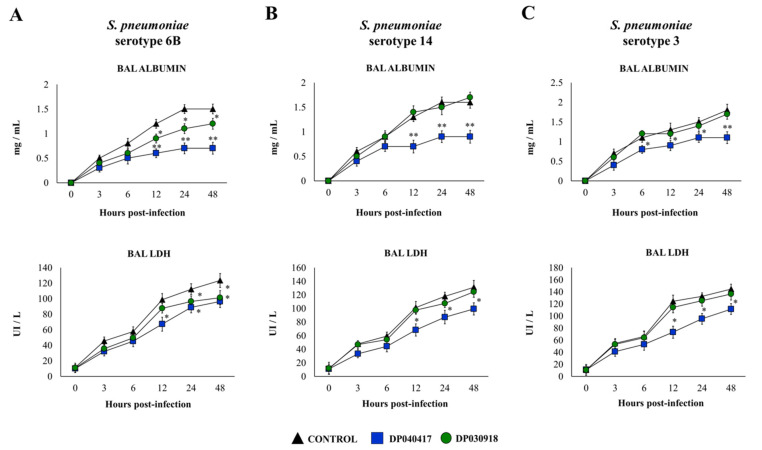
Effect of the respiratory commensal bacteria *Dolosigranulum pigrum* on resistance to *Streptococcus pneumoniae* infection. Infant mice were nasally primed with viable *D. pigrum* (DP) strain 040417 or 030918 during five consecutive days at a dose of 10^8^ bacterial cells/mouse/day. One day after the last administration of DP strains, mice were challenged with (**A**) *S. pneumoniae* serotypes 6B (**A**), 14 (**B**), or 3 (**C**). Non-treated infant mice challenged with the corresponding serotypes of *S. pneumoniae* were used as controls. Lactate dehydrogenase (LDH) activity and albumin concentrations in broncho-alveolar lavages (BAL) were evaluated on different time points after the bacterial challenge. The results represent data from three independent experiments (*n* = 6 per each group at each time point). Significantly different when compared with control at the same time point, * *p* < 0.05 or ** *p* < 0.01.

LDH and albumin in BAL samples were used as biochemical markers of lung tissue damage as described previously [20]. *S. pneumoniae*-infected mice showed a significant increase in the levels of both markers earlier after 3 h of the challenge with the three pneumococcal serotypes (Figure 2), indicating that the pathogens were capable of inducing cellular damage and alveolar-capillary barrier alterations. Both BAL albumin and LDH levels were significantly lower in infant mice treated with *D. pigrum* 040417 than in controls, while these BAL injury markers in *D. pigrum* 030918-treated infant mice were not different from the control mice (Figure 2).

### 3.2. D. pigrum 040417 Differentially Modulates the Respiratory Innate Immunity against S. pneumoniae

**Figure 3:**
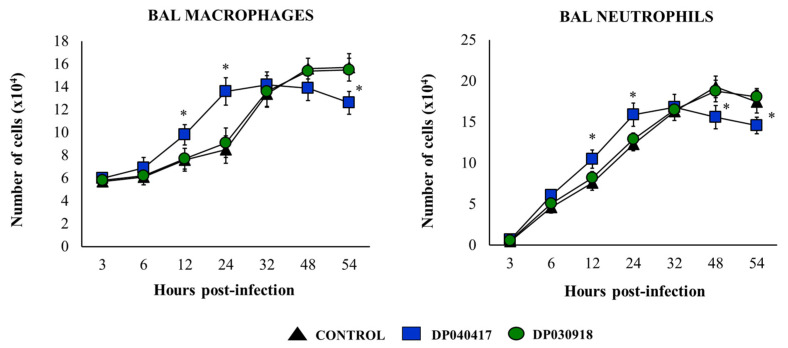
Effect of the respiratory commensal bacteria *Dolosigranulum pigrum* on resistance to *Streptococcus pneumoniae* infection. Infant mice were nasally primed with viable *D. pigrum* (DP) strain 040417 or 030918 during five consecutive days at a dose of 10^8^ bacterial cells/mouse/day. One day after the last administration of DP strains, mice were challenged with *S. pneumoniae* serotype 3. Non-treated infant mice challenged with *S. pneumoniae* were used as controls. Macrophages and neutrophils numbers in broncho-alveolar lavages (BAL) were evaluated on different time points after the bacterial challenge. The results represent data from three independent experiments (*n* = 6 per each group at each time point). Significantly different when compared with control at the same time point, * *p* < 0.05.

We next aimed to study the influence of *D. pigrum* on the respiratory innate immune response against the pneumococcal infection. *S. pneumoniae* serotype 3 was selected for further experiments, taking into consideration that it is one of the most virulent pneumococcal serotypes in mice. The variations in the numbers of immune cell populations in BAL after the pneumococcal infection were evaluated as shown in Figure 3. The challenge with *S. pneumoniae* significantly increased the numbers of BAL macrophages and neutrophils in the three experimental groups. The numbers of BAL macrophages as well as neutrophils counts in *D. pigrum* 030918-treated mice were not different from controls. Of note, *D. pigrum* 040417-treated mice had kinetics of BAL macrophages and neutrophils that were significantly different from controls (Figure 3). In 040417-treated mice, BAL macrophages and neutrophils were higher than controls in the first hours post-infection, while the numbers of these immune cells were lower than in controls from hour 48 post-infection.

**Figure 4:**
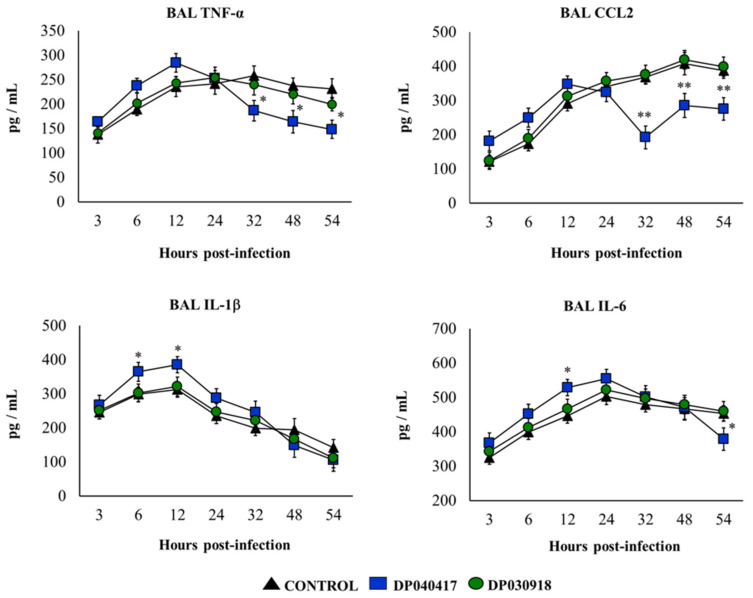
Effect of the respiratory commensal bacteria *Dolosigranulum pigrum* on resistance to *Streptococcus pneumoniae* infection. Infant mice were nasally primed with viable *D. pigrum* (DP) strain 040417 or 030918 during five consecutive days at a dose of 10^8^ bacterial cells/mouse/day. One day after the last administration of DP strains, mice were challenged with *S. pneumoniae* serotype 3. Non-treated infant mice challenged with *S. pneumoniae* were used as controls. Tumour necrosis factor (TNF)-α, chemokine (C–C motif) ligand 2 (CCL2), interleukin (IL)-1β, and IL-6 concentrations in broncho-alveolar lavages (BAL) were evaluated on different time points after the bacterial challenge. The results represent data from three independent experiments (*n* = 6 per each group at each time point). Significantly different when compared with control at the same time point, * *p* < 0.05 or ** *p* < 0.01.

We also evaluated the concentration of respiratory pro-inflammatory cytokines in BAL samples before and after the challenge with *S. pneumoniae*. The treatment of mice with *D. pigrum* 040417 or 030918 did not induce changes in the basal levels of BAL CCL2 or IL-1β compared with controls (Figure S1). In addition, no differences between controls and *D. pigrum* 030918-treated mice were found when the levels of BAL IL-6 were assessed in basal conditions. In contrast, the 040417 strain increased BAL IL-6 (Figure S1). In addition, we showed that both *D. pigrum* strains slightly enhanced BAL TNF-α (Figure S1). As shown in Figure 4, an early increase of BAL TNF-α, IL-1β, IL-6, and CCL2 was detected in all the experimental groups after the challenge with *S. pneumoniae*. In control mice, BAL TNF-α and IL-1β started to decrease after 24 h, while BAL IL-6 levels diminished from hour 32. In contrast, BAL CCL2 concentrations continued increasing during all the studied periods. The concentrations of BAL pro-inflammatory cytokines in *D. pigrum* 030918-treated mice were not different from controls (Figure 4). In contrast, mice treated with *D. pigrum* 040417 had significantly reduced levels of BAL TNF-α and CCL2 compared with controls after 32 hours post-infection. In addition, BAL IL-1β levels were significantly higher in mice that received the 040417 treatment compared with controls in the first hours post-infection (Figure 4). BAL IL-6 levels in *D. pigrum* 040417-treated mice were higher and lower than controls at hours 12 and 54, respectively.

**Figure 5:**
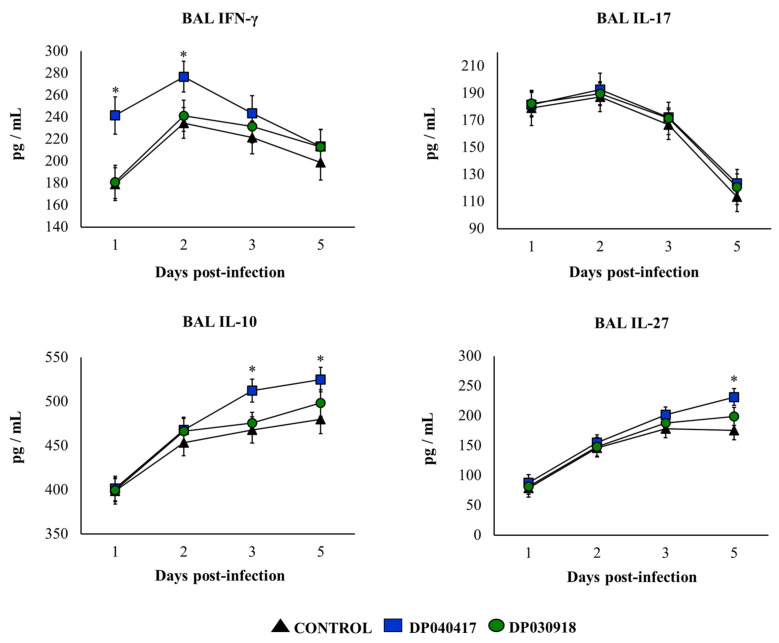
Effect of the respiratory commensal bacteria *Dolosigranulum pigrum* on resistance to *Streptococcus pneumoniae* infection. Infant mice were nasally primed with viable *D. pigrum* (DP) strain 040417 or 030918 during five consecutive days at a dose of 10^8^ bacterial cells/mouse/day. One day after the last administration of DP strains, mice were challenged with *S. pneumoniae* serotype 3. Non-treated infant mice challenged with *S. pneumoniae* were used as controls. Interferon (IFN)-γ, interleukin (IL)-17, IL-10, and IL-27 concentrations in broncho-alveolar lavages (BAL) were evaluated on different time points after the bacterial challenge. The results represent data from three independent experiments (*n* = 6 per each group at each time point). Significantly different when compared with control at the same time point, * *p* < 0.05.

We previously reported that *D. pigrum* 040417 can increase the levels of BAL IFN-γ and IL-10 in basal conditions, an effect that is not achieved by the 030918 strain [12]. We also evaluated here the effect of both respiratory commensal bacteria on the levels of BAL IL-17 and IL-27, and no differences were found when compared with control mice (data not shown). The concentrations of IFN-γ and IL-17 as well as of the immunoregulatory cytokines IL-10 and IL-27 were determined in BAL samples after the pneumococcal infection (Figure 5). The four cytokines were significantly increased after the challenge with *S. pneumoniae* in all the experimental groups. Both BAL IFN-γ and IL-17 showed a peak on day 2 post-infection and then started to decrease in all the groups. However, mice treated with *D. pigrum* 040417 had significantly higher levels of BAL IFN-γ than controls on days 1 and 2. In addition, the treatment with the strain 040417 allowed infant mice to produce higher levels of IL-10 from day 3 post-infection and IL-27 on day 5 (Figure 5). The concentrations of BAL cytokines in *D. pigrum* 030918-treated mice were not different from controls (Figure 5).

### 3.3. D. pigrum 040417 Differentially Modulates the Activation of Alveolar Macrophages

**Figure 6:**
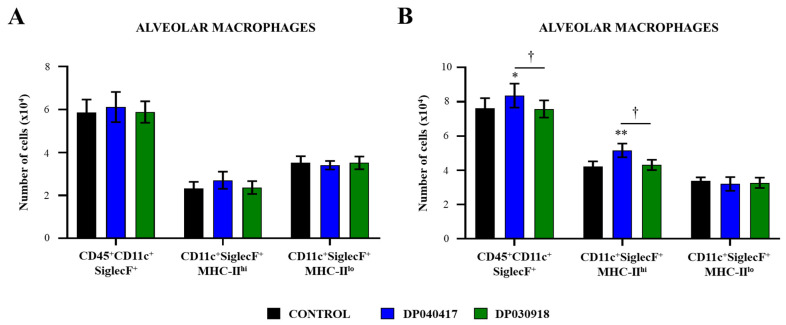
Effect of the respiratory commensal bacteria *Dolosigranulum pigrum* on resistance to *Streptococcus pneumoniae* infection. Infant mice were nasally primed with viable *D. pigrum* (DP) strain 040417 or 030918 during five consecutive days at a dose of 10^8^ bacterial cells/mouse/day. One day after the last administration of DP strains, mice were challenged with *S. pneumoniae* serotype 3. Non-treated infant mice challenged with *S. pneumoniae* were used as controls. Total resident alveolar macrophages populations in broncho-alveolar lavages (BAL) (CD45^+^CD11c^+^SiglecF^+^ cells) as well as their expression of MHC-II were evaluated by flow cytometry before (**A**) and 2 days after (**B**) the bacterial infection. The results represent data from three independent experiments (*n* = 6 per each group at each time point). Significantly different when compared with control at the same time point, * *p* < 0.05 or ** *p* < 0.01. Significantly differences between the indicated groups, † *p* < 0.05.

The variations of resident AMs in BAL samples were characterized by flow cytometry as described previously [21,22]. The BAL CD45^+^CD11c^+^SiglecF^+^ cells, corresponding to the total resident AMs population, were evaluated 1 day after treatments with the *D. pigrum* strains (Figure 6A), and 2 days after the infection with *S. pneumoniae* (Figure 6B). *D. pigrum* treatments were not able to modify the total number of AMs in the steady state. The challenge with *S. pneumoniae* increased the numbers of BAL CD45^+^CD11c^+^SiglecF^+^ cells in all the experimental groups; however, mice primed with *D. pigrum* 040417 had significantly higher numbers of macrophages compared with controls (Figure 6). No differences were found in BAL CD45^+^CD11c^+^SiglecF^+^ cells numbers when *D. pigrum* 030918-treated and control mice were compared after the pneumococcal infection.

The CD11c^+^SiglecF^+^MHC-II^hi^ AMs population was also evaluated in BAL samples. In control mice, the AMs with an activated phenotype represented around the 25% of the total resident AMs population, most macrophages being MHC-II^lo^ cells (Figure 6A). *D. pigrum* treatments were not able to modify the numbers of BAL CD11c^+^SiglecF^+^MHC-II^hi^ or CD11c^+^SiglecF^+^MHC-II^lo^ cells in the steady state. BAL CD11c^+^SiglecF^+^MHC-II^hi^ cells significantly increased after the bacterial infection in all the groups (Figure 6B). However, the treatment with *D. pigrum* 040417 enhanced the numbers of BAL CD11c^+^SiglecF^+^MHC-II^hi^ cells compared with controls. In contrast, mice treated with *D. pigrum* 030918 did not show statistically significant modifications in comparison with controls when the numbers of CD11c^+^SiglecF^+^MHC-II^hi^ cells after the challenge with *S. pneumoniae* were analysed. In addition, BAL CD11c^+^SiglecF^+^MHC-II^lo^ AMs in mice treated with both *D. pigrum* strains were not different from controls on day 2 post-infection (Figure 6B).

### 3.4. D. pigrum 040417 Modulates Alveolar Macrophages Cytokine Production

**Figure 7:**
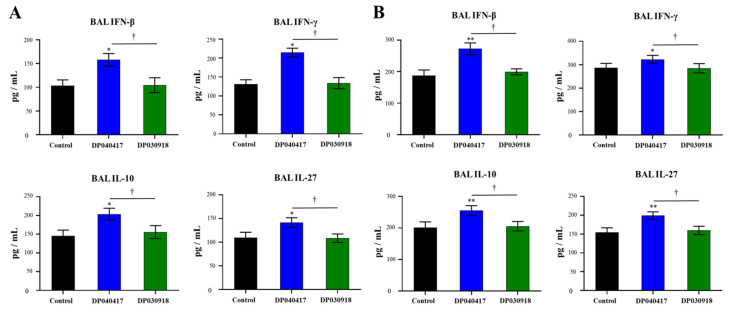
Effect of the respiratory commensal bacteria *Dolosigranulum pigrum* on the alveolar macrophages’ cytokine production. Infant mice were nasally primed with viable *D. pigrum* (DP) strain 040417 or 030918 during 5 consecutive days at a dose of 10^8^ bacterial cells/mouse/day. One day after the last administration of DP, alveolar macrophages were harvested and cultured for 24 h. The levels of interferon (IFN)-β, IFN-γ, interleukin (IL)-10, and IL-27 (pg/mL) in culture supernatants were determined after the 24 h of culture (**A**) or after 24 h of the in vitro challenge with *Streptococcus pneumoniae* (**B**). Cells from non-treated infant mice were used as controls. The results represent data from three independent experiments (*n* = 6 per each group at each time point). Significantly different when compared with control, * *p* < 0.05 or ** *p* < 0.01. Significantly differences between the indicated groups, † *p* < 0.05.

We recently demonstrated that cytokines produced by AMs play a relevant role in the immunomodulatory effect of nasally [21] or orally [22] administered beneficial bacteria. For this reason, we evaluated the influence of *D. pigrum* strains on the cytokine profile of AMs. Thus, primary cultures of AMs from control, *D. pigrum* 040417- and *D. pigrum* 030918-treated infant mice were performed and cells were cultured for 24 h. The basal production of IFN-β, IFN-γ, as well as of the immunoregulatory cytokines IL-10 and IL-27, was evaluated (Figure 7A). The four cytokines were detected in culture supernatants of AMs, in line with our previous reports [21,22]. Of note, the concentrations of IFN-β, IFN-γ, IL-10, and IL-27 were significantly higher in the AMs cultures obtained from infant mice treated with *D. pigrum* 040417 than in controls. In contrast, the levels of all the cytokines in supernatants of the AMs obtained from *D. pigrum* 030918-treated infant mice were not different from controls (Figure 7A).

In a similar set of experiments, AMs obtained from control, *D. pigrum* 040417- and *D. pigrum* 030918-treated infant mice were challenged in vitro with *S. pneumoniae* serotype 3, and the levels of IFN-β, IFN-γ, IL-10, and IL-27 in culture supernatants were assessed 24 h post-infection (Figure 7B). The pneumococcal challenge significantly increased the levels of the four cytokines in all the experimental groups compared with their respective basal levels. Of note, the concentrations of IFN-β, IFN-γ, IL-10, and IL-27 were significantly higher in AMs cultures from *D. pigrum* 040417-treated mice compared with the control group. No differences were observed in the levels of the four cytokines when AMs from *D. pigrum* 030918-treated mice were compared with those obtained from the control group after the pneumococcal challenge (Figure 7B).

### 3.5. The Depletion of Alveolar Macrophages Significantly Affects the Ability of D. pigrum 040417 to Protect against Pneumococcal Infection

**Figure 8:**
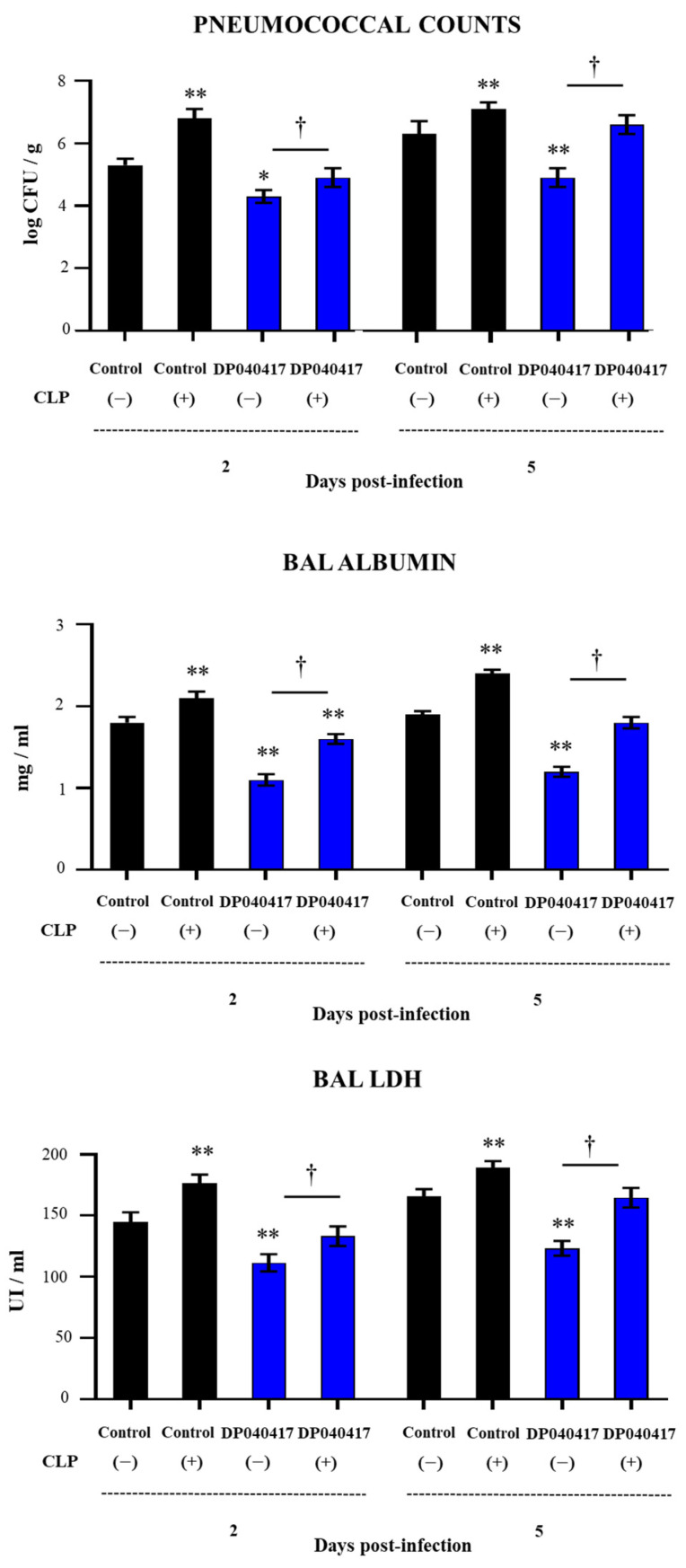
Effect of alveolar macrophages depletion on the ability of the respiratory commensal bacteria *Dolosigranulum pigrum* 040417 to improve protection against *Streptococcus pneumoniae* infection. Infant mice were nasally treated with clodronate-containing liposomes (CLP) during 2 days (days 0 and 1) to induce the depletion of alveolar macrophages. One day after the last CPL administration, mice were nasally treated with viable *D. pigrum* (DP) strain 040417 (10^8^ cell/mouse/day) during five consecutive days (days 2–6) and challenged with *S. pneumoniae* serotype 3 on day 7. Mice treated with liposomes and then challenged with *S. pneumoniae* were used as controls. Two and five days after the challenge with the pathogen, pneumococcal cell counts, lactate dehydrogenase (LDH) activity and albumin concentrations in broncho-alveolar lavages (BAL) were evaluated. The results represent data from three independent experiments (*n* = 6 per each group at each time point). Significantly different when compared with control CLP (−) group, * *p* < 0.05 or ** *p* < 0.01. Significantly differences between the indicated groups, † *p* < 0.05.

In order to evaluate further the role of AMs in the immunomodulatory effects of *D. pigrum* 040417 in the context of pneumococcal pneumonia, we depleted AMs using clodronate-containing liposomes (CLP). AMs were depleted before the stimulation of mice with the 040417 strain, and the mice were then challenged with *S. pneumoniae*. Two and five days after the pathogen challenge, pneumococcal cell counts (Figure 8) and the lung injury markers—BAL LDH activity and albumin concentration—were evaluated (Figure 8). Control mice treated with CLP and challenged with *S. pneumoniae* showed a higher susceptibility to the respiratory infection. In fact, the pulmonary colonization of the respiratory pathogen was significantly higher than the control group in the two time points post-infection evaluated (Figure 8). In addition, the levels of the biochemical markers of lung damage were significantly higher in control CLP-treated mice than in control animals (Figure 8). The ability of the *D. pigrum* 040417 treatment to reduce *S. pneumoniae* counts in the lungs and the levels of BAL albumin and LDH was abolished when AMs were depleted by CLP (Figure 8). Of note, lung pneumococcal cell counts and BAL LDH in CLP- and 040417-treated mice were not different from control animals.

**Figure 9:**
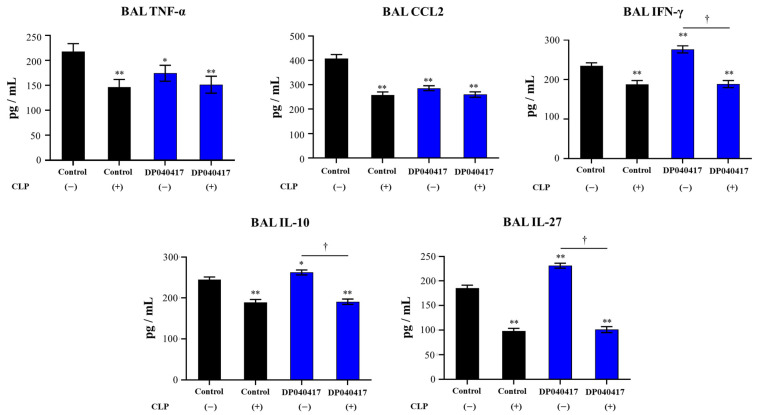
Effect of alveolar macrophages depletion on the ability of the respiratory commensal bacteria *Dolosigranulum pigrum* 040417 to improve protection against *Streptococcus pneumoniae* infection. Infant mice were nasally treated with clodronate-containing liposomes (CLP) during 2 days (days 0 and 1) to induce the depletion of alveolar macrophages. One day after the last CPL administration, mice were nasally treated with viable *D. pigrum* (DP) strain 040417 (10^8^ cell/mouse/day) during five consecutive days (days 2–6) and challenged with *S. pneumoniae* serotype 3 on day 7. Mice treated with liposomes and then challenged with *S. pneumoniae* were used as controls. Tumour necrosis factor (TNF)-α, chemokine (C–C motif) ligand 2 (CCL2), and interferon (IFN)-γ in broncho-alveolar lavages (BAL) were evaluated 2 days after the pathogen challenge. Interleukin (IL)-10 and IL-27 in BAL were evaluated 5 days after the pathogen challenge. The results represent data from three independent experiments (*n* = 6 per group). Significantly different when compared with control CLP (−) group, * *p* < 0.05 or ** *p* < 0.01. Significantly differences between the indicated groups, † *p* < 0.05.

Finally, we evaluated the effect of AMs depletion on the levels of BAL cytokines in response to pneumococcal infection and the influence of *D. pigrum* treatment. As shown in Figure 9, the concentrations of BAL TNF-α, CCL2, IFN-γ, IL-10, and IL-27 in infant mice treated with CLP were significantly lower than those observed in control animals. In addition, it was observed that CLP treatment significantly affected the ability of *D. pigrum* 040417 to differentially modulate the BAL cytokine profile in response to pneumococcal infection (Figure 9). The concentrations of BAL TNF-α and CCL2 were not affected when CLP were administered to mice receiving the 040417 strain. In fact, the levels of both cytokines were similar to those found in both control CLP-treated mice and control animals. On the other hand, the ability of *D. pigrum* 040417 treatment to increase the levels of BAL IFN-γ, IL-10, and IL-27 was abolished when AMs were depleted by CLP. Of note, the concentrations of these three BAL cytokines in CLP- and 040417-treated mice were not different from CLP control animals.

## 4. Discussion

Recent research demonstrated that *D. pigrum* is one of the main beneficial members of the respiratory microbiota as evidence suggests that this species of bacteria plays a protective role in the respiratory mucosa [6,7]. Although the abundance of *D. pigrum* in the URT was shown to be associated with a low-density colonization in the context of pneumococcal exposure in adults [23], most of the studies that reported benefits on respiratory health induced by this bacterium have been conducted in children [7,8,9,24]. The abundance of *D. pigrum* in the URT was shown to be inversely associated with respiratory tract infections and wheezing in children [24]. Furthermore, in paediatric populations where *S. pneumoniae* is absent, *D. pigrum* is overrepresented [8]. In fact, children with *D. pigrum* colonization of the URT are less likely to be colonized with *S. pneumoniae* [8,9] and have reduced risk of suffering from acute otitis media [9,25] or invasive pneumococcal disease [7].

Studies have begun to elucidate the mechanisms by which *D. pigrum* may improve the resistance to pneumococcal infection. It was shown that the microbe–microbe interaction explains, at least partially, this phenomenon. It was proposed that the local production of lactic acid by *D. pigrum* would account for epidemiologic observations of negative associations between *S. pneumoniae* and this commensal respiratory bacterium [26]. However, Brugger et al. [10] have recently challenged this hypothesis and demonstrated that the production of lactic acid is not a unique contributor to the restriction of pneumococci’s growth by *D. pigrum*. Authors proposed that the competition for nutrients and the production of a diffusible inhibitory compounds accounted for the inhibition of *S. pneumoniae*. In fact, the comparative genomic analysis of several *D. pigrum* strains demonstrated the presence of a diversity of biosynthetic gene clusters predicted to encode candidate antibiotic-like compounds [10]. Here we propose a different alternative—not mutually exclusive with the hypothesis of direct microbe–microbe interaction—which is the indirect effect through the modulation of the immune system. Our results indicate that the *D. pigrum*–host immune system interaction can increase resistance to the pneumococcal respiratory infection.

In this work, we have advanced in the characterization of the beneficial properties of *D. pigrum* 040417 by studying its capacity to modulate the respiratory innate immune response triggered by pneumococcal infection in infant mice. In our hands, the nasal priming with the 040417 strain significantly reduced pneumococcal cell counts, avoided dissemination into blood and diminished lung tissue injuries. Interestingly, *D. pigrum* 040417 was able to increase the resistance to serotypes 6B, 14, and 3 indicating that the stimulation of the respiratory innate immune response induced by this commensal respiratory bacterium can increase the resistance to different pneumococcal serotypes. This undoubtedly represents an advantage in the prevention of pneumococcal infections because one of the most important obstacles in this regard is achieving serotype-independent protection. Our results showed a significant improvement in the respiratory innate immune response in infant mice treated with *D. pigrum* 040417. This was reflected in the induction of an enhanced inflammatory response in the early stages of infection characterized by increases in the numbers of macrophages and neutrophils as well as increases in the levels of IL-1β, IFN-γ, and IL-6. On the other hand, this inflammatory response was more efficiently regulated in the later stages of the infection, as evidenced by decreased levels of neutrophils, TNF-α, CCL2, and IL-6 and the increases in the regulatory cytokines IL-10 and IL-27. Thus, the different magnitude and kinetics of the *D. pigrum* 040417-induced innate immune response in the respiratory tract probably explains the decrease in pneumococcal colonization and the protection against the inflammatory lung damage.

AMs are the most abundant innate immune cells in the lower respiratory tract. This immune cell population is the first to face pathogenic bacteria that reach the deep lung, and has a key role in the generation and regulation of immune responses against those pathogens [27]. Here, we demonstrated for the first time that AMs have a relevant role in the immunomodulatory effect of *D. pigrum* 040417. Our in vitro experiments showed that AMs obtained from 040417-treated infant mice were able to produce higher levels of IFN-β and IFN-γ, two cytokines that were associated to the protection against pneumococcal infection. It was reported that improved levels of IFN-γ are critical for host defence against *S. pneumoniae* infection because of its ability to stimulate AMs [28]. In this regard, transcriptomic analysis of whole lungs of mice infected with *S. pneumoniae* revealed that the up-regulation of IFN-γ and IFN-related genes was associated with the protection against this respiratory pathogen [29]. On the other hand, it was shown that AMs produce type I IFNs upon the in vitro challenge with *S. pneumoniae* via a bacterial uptake-dependent mechanism. Furthermore, in vivo studies confirmed that AMs are the main source of IFN-α and IFN-β upon the pneumococcal challenge [30]. The production of type I IFNs by AMs modulates alveolar type II pneumocytes, increasing their resistance to the pneumococcal infection and protecting them from cell death [30]. Interestingly, IFN-β was associated with the control of pneumococcal dissemination into the blood because pneumococci were observed earlier and at higher numbers in blood samples of IFNAR1^−/−^ mice compared with wild type animals [31]. In addition, nasally delivered IFN-β was capable of enhancing the protection of mice against pneumococcal systemic disease [31]. These previous results are in line with our data demonstrating that *D. pigrum* 040417-treated infant mice have no detectable pneumococcal cells in haemocultures at none of the studied time points.

Our results also indicated that AMs play an important role in the protective effect of *D. pigrum* 040417 against the lung inflammatory damage produced during pneumococcal infection. The regulation of excessive inflammation is a key factor in the outcome of *S. pneumoniae* infections [32]. It was shown that AMs possess the ability to promote Treg cell responses by directly interacting with these cells or indirectly, through the production of cytokines [33,34]. We demonstrated previously that *D. pigrum* 040417 increased lung CD3^+^CD4^+^IL-10^+^ T cells in the respiratory tract of infant mice [12]. Here we showed that AMs from infant mice nasally primed with the 040417 strain had a significantly enhanced ability to produce IL-10 and IL-27 in response to *S. pneumoniae* infection, which was in line with the in vivo determinations of both regulatory cytokines. Of note, it was demonstrated that IL-27 is not sufficient for the optimal induction of Treg cell maturation in the respiratory tract and that IL-6 is required for the IL-27/Treg cell protections against inflammatory damage [21,33]. Interestingly, mice treated with *D. pigrum* 040417 had a differential production of IL-6 during pneumococcal infection compared with infected controls. Our data thus indicate that the improved production of IL-10, IL-27, and IL-6 by AMs of 040417-treated infant mice may play an important role in limiting inflammation during the pneumococcal infection by increasing the protective functions of Treg cells.

The depletion of AMs by clodronate liposomes at the time of *D. pigrum* 040417 nasal priming allowed us to confirm the important role of this respiratory immune cell population in the protective effect of the respiratory commensal bacterium. The improved production of IFN-γ, IL-10, and IL-27 in the respiratory tract of 040417-treated mice in response to the pneumococcal challenge was reduced in CLP-treated animals. Furthermore, the capacity of *D. pigrum* 040417 to reduce pneumococcal cell counts as well as the biomarkers of lung injury was significantly affected when AMs were depleted.

The concept of “trained immunity” implies that innate immune cells, such as macrophages, can be modified, after a primary immunologic challenge, to carry a nonspecific immune memory, which improves their responses to subsequent related or unrelated immunologic exposures [35,36]. Trained immunity was evaluated in the context of respiratory infections [36,37] and it was demonstrated that AMs with a trained immunity phenotype had enhanced expression of MHC-II and release of cytokines and chemokines upon restimulation [37]. Of note, IFN-γ production during the primary immunologic challenge was associated to the generation of trained AMs [35,36,37]. In line with those studies, we demonstrated recently that nasally administered nonviable *Lacticaseibacillus rhamnosus* CRL1505 modulated respiratory immunity, protected against bacterial and viral pathogens, and that the generation of activated/trained AMs was essential to achieve such beneficial effects [21]. Our studies focused on resident CD45^+^CD11c^+^SiglecF^+^ AMs demonstrated that the nasal priming with nonviable *L. rhamnosus* CRL1505 significantly increased their expression of MHC-II 2 days after the RSV infection, which corresponded to 7 days after the CRL1505 treatment [21]. This was consistent with reports describing that trained CD11c^+^CD64^+^SiglecF^+^ AMs began to develop between 5–7 days after the primary immunologic challenge [37]. In addition, AMs from *L. rhamnosus* CRL1505-treated infant mice had an enhanced in vitro production of IFN-γ and IL-6, which was correlated with an improved immune response in vivo upon a secondary pneumococcal challenge. Similarly, we reported here that the nasal treatment with *D. pigrum* 040417 increased the numbers of BAL CD11c^+^SiglecF^+^MHC-II^hi^ cells and enhanced the cytokine response of AMs after the in vitro pneumococcal challenge. It is thus tempting to speculate that bacterial treatments, such as with nonviable *L. rhamnosus* CRL1505 or viable *D. pigrum* 040417, are able to promote trained immunity in AMs and, in this way, to increase protection against respiratory pathogens. However, it should be considered that induction of a trained immune phenotype in macrophages involves an epigenetic reprogramming that enables them to react with a faster, stronger, and/or qualitatively different transcriptional response after subsequent immunologic challenge [38]. Therefore, further transcriptomic and epigenetic studies are necessary to conclusively assert that *L. rhamnosus* CRL1505 or *D. pigrum* 040417 beneficially modulate respiratory immunity through the promotion of trained immunity in AMs.

One of the most relevant characteristics of probiotics is that their beneficial properties depend on each particular strain, in such a way that, for example, the immunomodulatory activity of one microorganism cannot be extrapolated to others, not even of the same species [39,40]. In this regard, our recently published results demonstrated for the first time the strain-dependent ability of *D. pigrum* to modulate immunity in the respiratory tract [12]. Using a mixture of the TLR2 ligands MALP2 and Pam3CSK4 to mimic the respiratory pro-inflammatory response induced by Gram-positive bacterial infections, we demonstrated *D. pigrum* 040417 enhanced the levels IFN-γ, IFN-β, and IL-10 in the respiratory tract and reduced the lung injury markers after the activation of TLR2 in infant mice. This effect was not achieved by the nasal treatment of infant mice with *D. pigrum* 030918. In line with those previous findings, we demonstrated here that the 030918 strain was not capable of modulating the innate immune response triggered by the pneumococcal infection or protecting against *S. pneumoniae* lung colonization and dissemination into blood. Interestingly, the genomic comparison of *D. pigrum* strains revealed the existence of diverse repertoires of biosynthetic gene clusters coding for lanthipeptides and bacteriocins [10]. This differential genetic endowment was associated to the distinct ability of *D. pigrum* strains to inhibit *Staphylococcus aureus* growth. These and our results indicate that genetic variability between different strains of *D. pigrum* could be a beneficial property for the host, since different strains may contribute to the protection against respiratory pathogens through different mechanisms that complement and enhance each other.

The recent understanding of how the mucosal immune system cooperates with microbes that colonize the respiratory tract to maintain lung homeostasis and protection against pathogens holds considerable expectation for new approaches to modulate immune networks to prevent infections. Our results mark a significant advance in the positioning of *D. pigrum* as a next-generation probiotic for the respiratory tract and encourage further research to help in the development of non-antibiotic therapeutical approaches to enhance the prevention and treatment of pneumonia by using beneficial respiratory commensal bacteria.

## Data Availability

All the data related to this project are presented here.

## Supplementary Materials

The following are available online at https://www.mdpi.com/article/10.3390/microorganisms9061324/s1. Figure S1: Effect of the respiratory commensal bacteria *Dolosigranulum pigrum* on respiratory cytokines. Infant mice were nasally primed with viable *D. pigrum* (DP) strain 040417 or 030918 during 5 consecutive days. Non-treated infant mice were used as controls. TNF-α, CCL2, IL-1β, and IL-6 concentrations in broncho-alveolar lavages (BAL) were evaluated. Significantly different compared with control, * *p* < 0.05 or ** *p* < 0.01. Significantly differences between the indicated groups, † *p* < 0.05.
