# Supplementary figures and images for: The Respiratory Commensal Bacterium Dolosigranulum pigrum 040417 Improves the Innate Immune Response to Streptococcus pneumoniae
Source: Microorganisms. 2021 Jun 18;9(6):1324. doi: 10.3390/microorganisms9061324 (PMC8234606; doi:10.3390/microorganisms9061324)

**BAL IL-1 $\beta$**

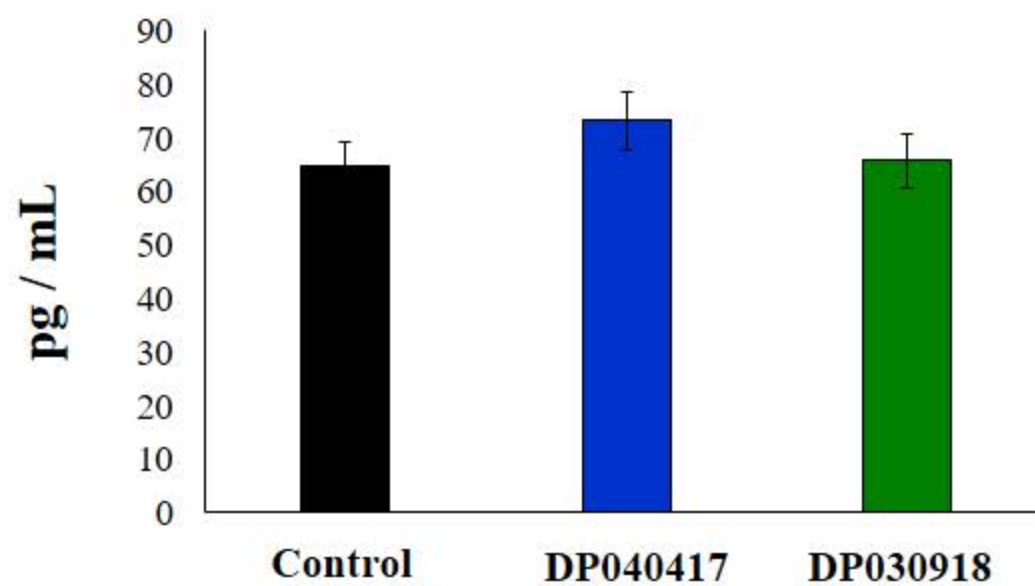

**BAL IL-6**

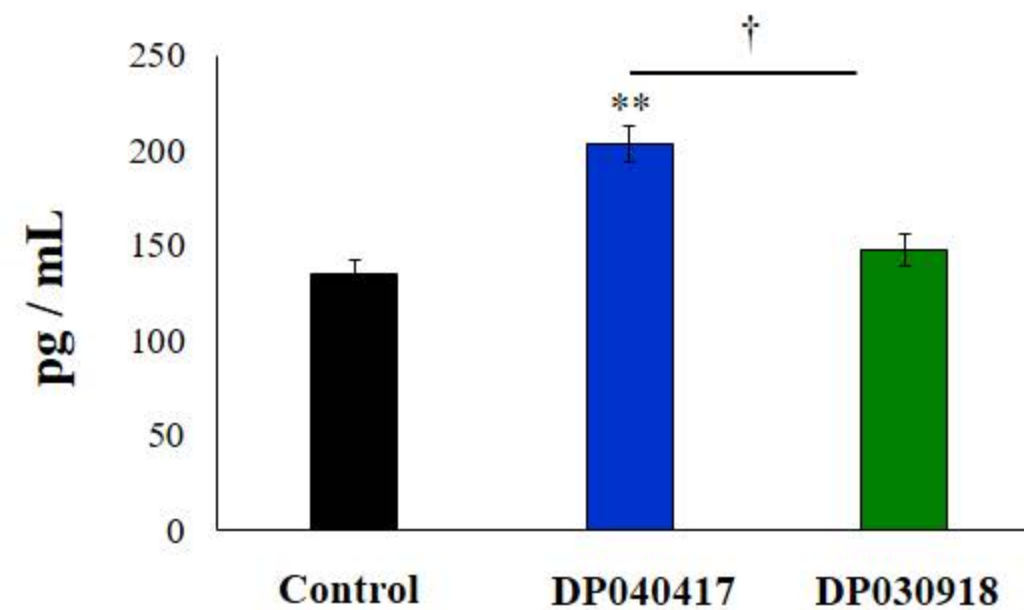

**BAL CCL2**

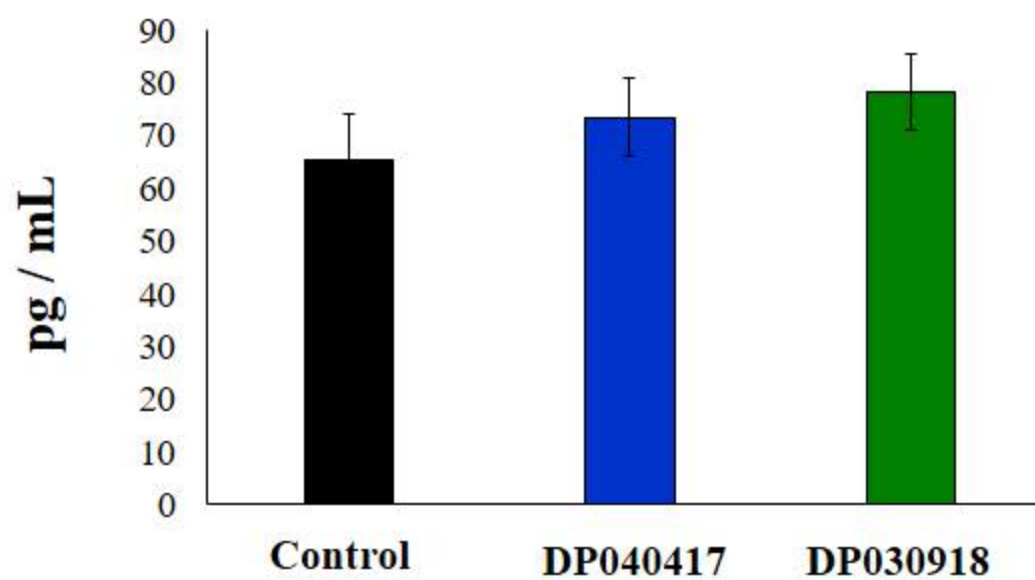

**BAL TNF- $\alpha$**

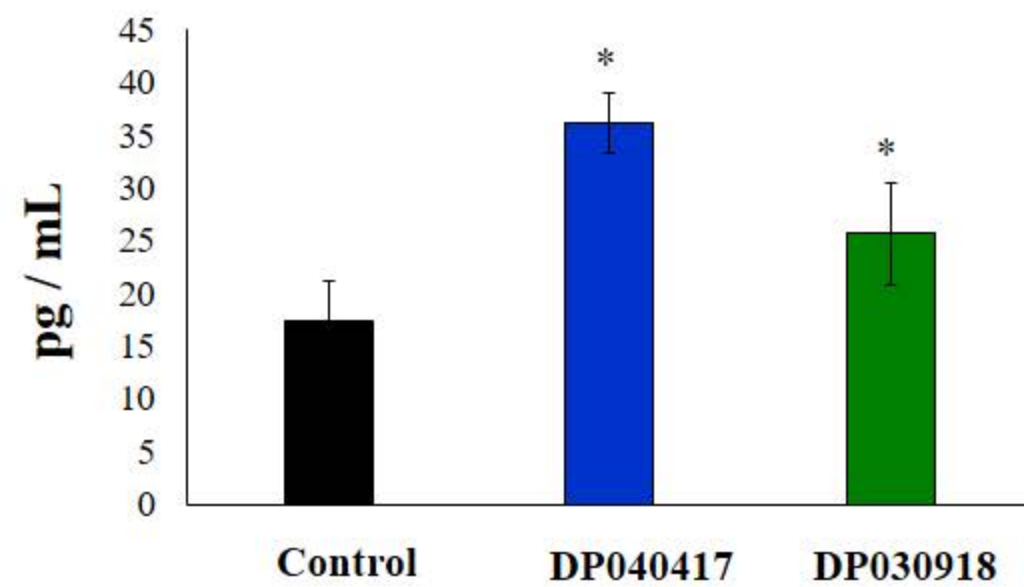

Supplement: Supplementary file 1 [file microorganisms-09-01324-s001.zip › microorganisms-1250778-supplementary.pdf]
